# Formulation of Novel Liquid Crystal (LC) Formulations with Skin-Permeation-Enhancing Abilities of *Plantago lanceolata* (PL) Extract and Their Assessment on HaCaT Cells

**DOI:** 10.3390/molecules26041023

**Published:** 2021-02-15

**Authors:** Dóra Kósa, Ágota Pető, Ferenc Fenyvesi, Judit Váradi, Miklós Vecsernyés, Sándor Gonda, Gábor Vasas, Pálma Fehér, Ildikó Bácskay, Zoltán Ujhelyi

**Affiliations:** 1 Department of Pharmaceutical Technology, Faculty of Pharmacy, University of Debrecen, Nagyerdei körút 98, 4032 Debrecen, Hungary; kosa.dora@pharm.unideb.hu (D.K.); peto.agota@pharm.unideb.hu (Á.P.); fenyvesi.ferenc@pharm.unideb.hu (F.F.); varadi.judit@pharm.unideb.hu (J.V.); vecsernyes.miklos@pharm.unideb.hu (M.V.); feher.palma@pharm.unideb.hu (P.F.); ildiko.bacskay@pharm.unideb.hu (I.B.); 2 Doctoral School of Pharmaceutical Sciences, University of Debrecen, Nagyerdei körút 98, 4032 Debrecen, Hungary; 3 Institute of Healthcare Industry, University of Debrecen, Nagyerdei körút 98, 4032 Debrecen, Hungary; 4 Department of Botany, Faculty of Science and Technology, University of Debrecen, Nagyerdei körút 98, 4032 Debrecen, Hungary; gonda.sandor@science.unideb.hu (S.G.); vasas.gabor@science.unideb.hu (G.V.)

**Keywords:** liquid crystals, *Plantago lanceolata*, penetration enhancers, antioxidant and anti-inflammatory effects, DPPH test, cytotoxicity investigation, MTT test, ROS, MDA, RTCA, TEER, HaCaT cells

## Abstract

Exposure to reactive oxygen species can easily result in serious diseases, such as hyperproliferative skin disorders or skin cancer. Herbal extracts are widely used as antioxidant sources in different compositions. The importance of antioxidant therapy in inflammatory conditions has increased. Innovative formulations can be used to improve the effects of these phytopharmacons. The bioactive compounds of *Plantago lanceolata* (PL) possess different effects, such as anti-inflammatory, antioxidant, and bactericidal pharmacological effects. The objective of this study was to formulate novel liquid crystal (LC) compositions to protect *Plantago lanceolata* extract from hydrolysis and to improve its effect. Since safety is an important aspect of pharmaceutical formulations, the biological properties of applied excipients and blends were evaluated using assorted in vitro methods on HaCaT cells. According to the antecedent toxicity screening evaluation, three surfactants were selected (Gelucire 44/14, Labrasol, and Lauroglycol 90) for the formulation. The dissolution rate of PL from the PL-LC systems was evaluated using a Franz diffusion chamber apparatus. The antioxidant properties of the PL-LC systems were evaluated with 2,2-diphenyl-1-picrylhydrazyl (DPPH) and malondialdehyde (MDA) assessments. Our results suggest that these compositions use a nontraditional, rapid-permeation pathway for the delivery of drugs, as the applied penetration enhancers reversibly alter the barrier properties of the outer stratum corneum. These excipients can be safe and highly tolerable thus, they could improve the patient’s experience and promote adherence.

## 1. Introduction

Human skin is our main protective organ against multiple environmental insults, such as reactive oxygen species (ROS) [1]. Although the exact method of the pathogenic effect of ROS is not fully understood, their role in serious diseases, such as hyperproliferative skin disorders or skin cancer, were clearly concluded [2]. Many efforts were directed toward reducing the source of ROS or neutralizing them [3]. Exposure to UV rays with 200–280 nm wavelength (UV-C) could result in frequent generation of reactive oxygen species [4]. UV-C has the ability to ionize, thus acting as a strong mutagen, which can cause the previously mentioned immune-mediated diseases and cancer in adverse cases. Due to the increasing ordinary UV-C exposure, source reduction seems to be an impossible mission. According to this conclusion, robust and effective antioxidant therapy seems to be the only vital tool in this contest. 

Medical plants play an essential role in human health, as their chemical substances have beneficial therapeutic effects [5]. *Plantago lanceolata* (PL) is one of the most important species within the *Plantago* genus, which contains approximately 275 species all over the world [6]. The bioactive components of *P. lanceolata* possess various pharmacological properties, including anti-inflammatory, diuretic, antibacterial [7], hepatoprotective [8], and antioxidant [9] properties. *P. lanceolata* is currently used to treat throat and upper respiratory system conditions and is used topically for skin diseases as well [10]. It is also used in traditional medicine in the treatment of wounds, burns, and bleeding [11].

The main active pharmaceutical component of *P. lanceolata* is verbascoside (acteoside), which belongs to the phenylethanoid glycosides [12]. Although it has an excellent and well-known safety profile, its poor chemical stability limits its formulations due to hydrolysis [13]. Despite several advantageous effects, the high hydrophilicity of acteoside limits the range of possible applications [14]. To overcome these limitations, we decided to prepare liquid crystals to protect *Plantago lanceolata* extract from hydrolysis and to improve its effects. 

Liquid crystals are molecules with noncovalent bonds and various phases (mesophase) with different molecular orientations [15]. They are anisotropic materials that show the properties of both liquids and solids and have solubilizing capability for both oil- and water-soluble compounds [16]. They are usually formed from water and possibly oil by adding one or two surfactants within a definite concentration and temperature range [17]. Since a well-defined group of surfactants has an unambiguous penetration-enhancer effect, prudent choice of these excipients might counteract the adverse effect on antioxidant compositions. Chemical-induced cell function alteration can be advantageous and exploitable as well. The mechanism of penetration enhancers can be understood and limitation factors can be determined under in vitro conditions. The impact of these compounds on junctional protein (ZO-1, claudin-1, β-catenin, etc.) resorption and consequentially improved paracellular integrity was previously described [18]. The degree of efficacy depends on the physical properties and the chemical structures of these excipients and compositions [19]. In the case of similar structures when the hydrophilic head is changed from polyethylene glycol to propylene glycol, the main determining factor of cytotoxicity could be the monoester content and the length of the carbon chain. According to previous studies, the optimum chain length is between C8 and C12 [20]. However, chain length is not the only factor determining the increase in intestinal absorption; some other structural parameters, such as the degree of esterification, the position of unsaturation, and the nature of substituents, can also influence surfactants to act as penetration enhancers [21]. 

The objective of this study was to formulate safe dosage forms containing *Plantago lanceolata* extract with enhanced cutaneous drug delivery. In topical drug delivery, we face two major challenges. Besides the drug solubilization and adequate penetration of the stratum corneum, patient adherence to treatment is also important due to the particularly low compliance in topical drug administration. Pharmaceutical excipients can help address these challenges [22]. 

The aim of this study was to formulate and investigate novel PL-loaded liquid crystal (LC) delivery systems. Besides the active pharmaceutical ingredient (API) protective effect of the applied vehicle, the objective of the LC formulation was increased bioavailability. According to the preformulation results, four drug delivery formulations were selected with three penetration enhancers (Labrasol, Lauroglycol 90, and Gelucire 44/14) [23]. Since safety is an important aspect of pharmaceutical formulations, the biological properties of the applied excipients and blends were evaluated using real-time cell electric sensing (RTCA) and 3-(4,5-dimethylthiazol-2-yl)-2,5-diphenyltetrazolium bromide (MTT) cell viability tests on HaCaT human keratinocytes [24]. This in vitro model is widely used in scientific research studies for the rapid screening of the cytotoxicity of topically administered drugs [25]. As an in vitro model of hyperproliferative skin disorders, IL-1- and TNF-α-induced keratinocytes were cultured and tested as well [26]. To evaluate the effect of the formulated PL-LC systems, UV-C-induced oxidative stress was induced in every HaCaT cell type. The antioxidant properties of the drug delivery systems were investigated using the malondialdehyde (MDA) and 2,2-diphenyl-1-picrylhydrazyl (DPPH) methods. Our work demonstrates not only the efficiency differences between preparations with various penetration enhancers, but also their benefits in hyperproliferative skin disorder applications.

## 2. Results

### 2.1. Formulation of Liquid Crystals

**Figure 1:**
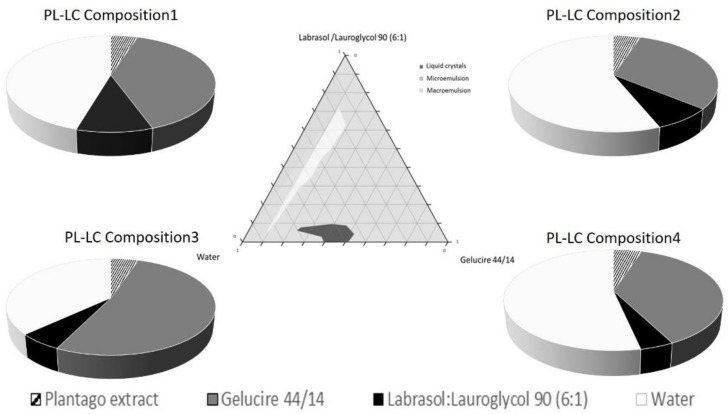
Pseudoternary phase diagram of the Labrasol/Lauroglycol 90 (6:1)/Gelucire 44/14/water system; the dark shaded area shows the liquid crystal (LC) zone. Component ratio of selected *Plantago lanceolata* (PL)-LC compositions (I–IV).

The aim of the pseudoternary phase diagram construction was to determine the occurrence range of the liquid crystals. The pseudoternary phase diagram of Labrasol/Lauroglycol 90 (6:1)/Gelucire 44/14/water is presented in Figure 1. To study the effect of water content on the liquid crystal structure, samples with different water contents (10–90%) were prepared. Mainly during the titration, microemulsions formed at various proportions of components. When the ratio of water was increased to above 90%, macroemulsions began to form, regardless of the proportion of Gelucire 44/14 and Labrasol/Lauroglycol 90 surfactants. In contrast, liquid crystal systems formed only at a certain, tighter range of the components when the mix contained Labrasol/Lauroglycol 90 surfactants under 10%. Four different compositions were selected according to the phase diagram with 6:1 constant ratio of Labrasol/Lauroglycol 90. Our systems were stable and clear in the Labrasol/Lauroglycol 90 range of 4.1–9.9% and the Gelucire 44/14 range 31.1–52%. The selected PL compositions are shown in Table 1.

### 2.2. In Vitro Permeation Study

**Figure 2:**
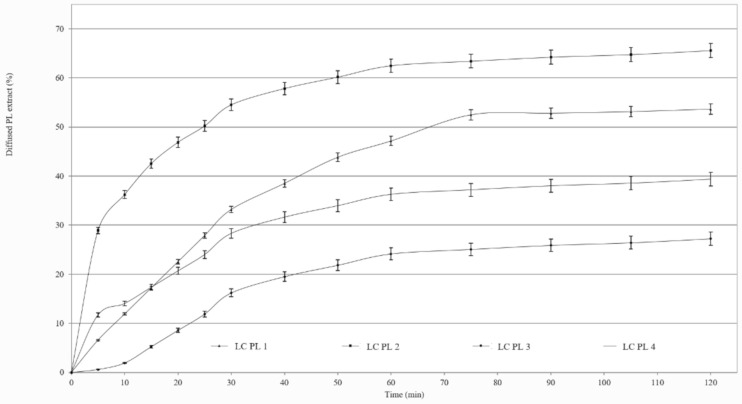
Release profiles of PL across isopropyl-myristate-impregnated cellulose acetate membrane of compositions I–IV.

Figure 2 shows the percentage of diffused PL extract through the synthetic cellulose acetate membrane from the formulations as a function of time. We ranked the release of drug from the four compositions in the following descending order: PL-LC 2 > 1 > 4 > 3. Our results showed that the drug release was better from the compositions that contained more of the surfactant pair Labrasol and Lauroglycol 90. The best diffusion was achieved from compositions I and II, in which the surfactant range was 9.9% and 7.8%, and the diffused amount of PL extract was over 50%. In the case of compositions III and IV, which contained less of the surfactants, worse drug diffusion occurred, as the released drug was under 40%.

### 2.3. Assessment of HaCaT Cell Proliferation

**Figure 3:**
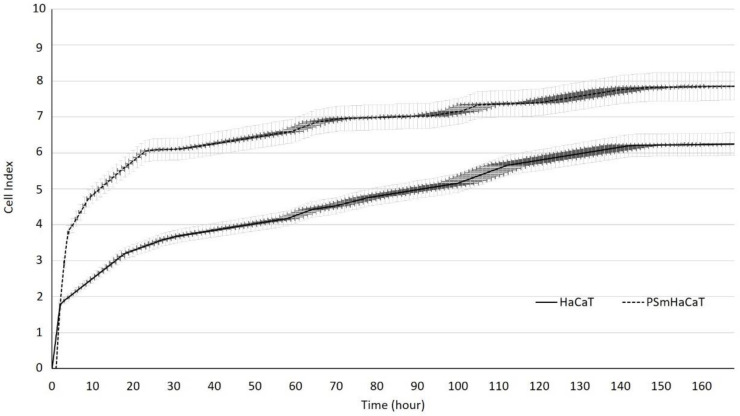
Impedance-based determination of HaCaT cell proliferation. Each data point represents the mean ± SD, *n* = 10.

**Figure 4:**
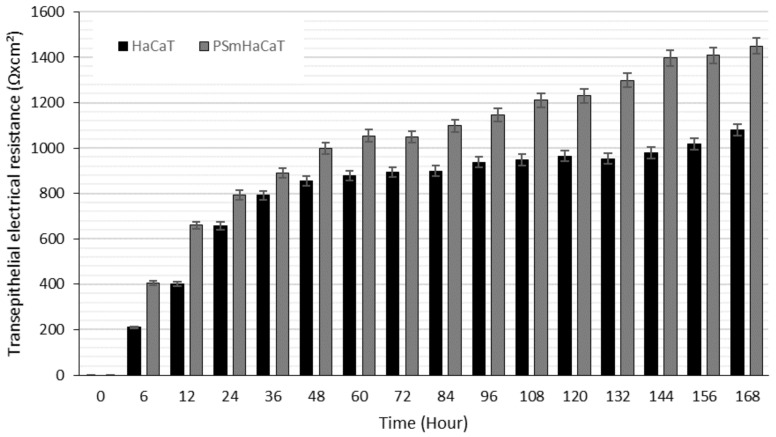
Transepithelial electrical resistance (TEER) measurements. Each data point represents the mean ± SD, *n* = 5.

Proliferation tests were conducted on HaCaT cells seeded at 10^4^/200 µL density using an xCELLigence Real-Time Cell Analyzer (RTCA). Figure 3 demonstrates the significant differences between the investigated samples. However, impedance-based determination of HaCaT cell proliferation demonstrated an obvious increase in the cell index in each of the cases, and the kinetics of the proliferation showed differences. Whereas the native HaCaT cells reached the confluent monolayer state after seven days, the IL-1β- and TNF-α-stimulated keratinocytes (PSmHaCaT) reached the plateau phase earlier after rapid proliferation. Moreover, PSmHaCaT keratinocytes demonstrated higher cell index values at this phase. To confirm this conclusion, classic transepithelial electrical resistance (TEER) measurements were also performed. The result of the transepithelial electrical resistance (TEER) measurements agreed with the RTCA tests. As shown in Figure 4, higher TEER values were observed in PSmHaCaT cells than in native keratinocytes at the same time.

### 2.4. MTT Viability Assay

**Figure 5:**
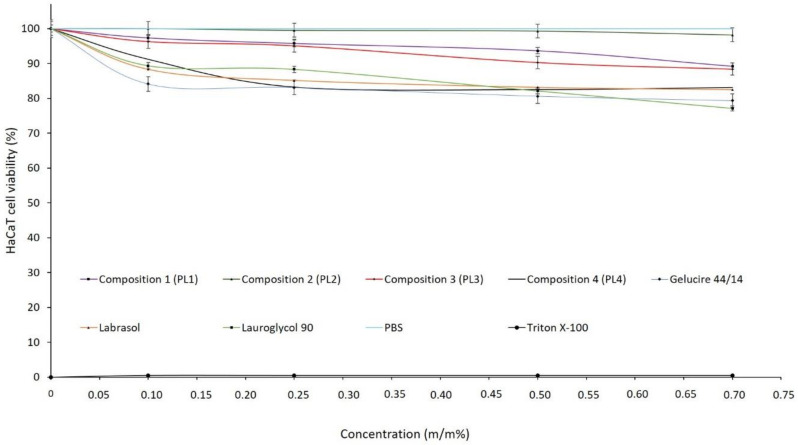
Toxic effects of formulated blends and their components on HaCaT cell viability. Each data point represents the mean ± SD, *n* = 10. The Triton-X-treated group was evaluated as the positive control; phosphate-buffered solution (PBS) was used as the negative control.

MTT cytotoxicity tests were performed on HaCaT cell monolayers to ensure safe application. The cytotoxicities of the formulated blends were evaluated in different concentrations. Each component of the compositions was also tested. Figure 5 shows that neither the formulated LC systems nor their components considerably affected the cell viability. The cell viability was less than 50% compared to that of the negative control (phosphate buffered saline (PBS)), even in a high concentration. We found that all compositions were tolerated well. There were no significant differences between LC- and PBS-treated (negative control) groups, although we concluded that composition II decreased cell viability the least.

### 2.5. DPPH Radical Scavenging Activity

**Figure 6:**
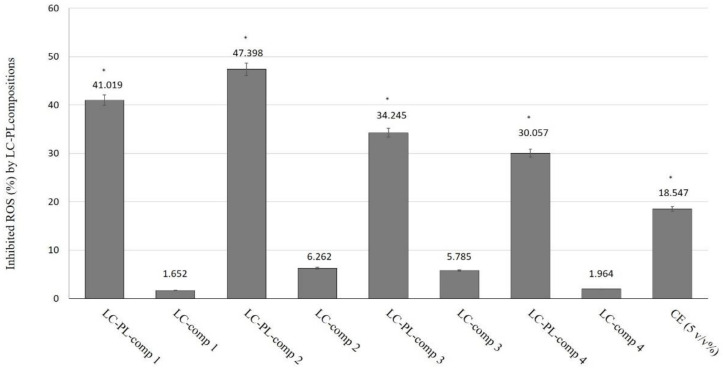
Inhibition of ROS (%) by different liquid crystal-Plantago Lanceolata extract (LC-PL) compositions. The positive control (CE) was water-dispersed P. lanceolata extract in 5% *v*/*v* concentration. The negative controls were the empty liquid crystal (LC) compositions. Values are expressed as means ± SD, *n* = 5. Significant differences between LC-PL-composition groups and the positive control group are marked with an asterisk (*).

The radical scavenging activity assay is based on the ability of a stable free radical (DPPH) to change color in the presence of antioxidants. The antioxidant capacities of LC compositions I–IV, with or without PL extracts, were tested. As controls, the appropriate blank LC formulations and water dispersion of 5% PL extract (CE) were applied. As previously described, formulated LC samples also contained 5% *v/v* PL extract. The percentage of antioxidant activity (AA%) of each substance was assessed using a DPPH free radical assay. The DPPH radical scavenging activity was measured according to the methodology described by Brand-Williams et al. [27]. According to our results, we concluded that there were significant differences between LC-PL-composition-treated groups, compositions without PL- and PL-composition-treated groups, and the CE-treated positive control sample. Significant differences are shown in Figure 6, marked with an asterisk. The performed radical scavenging activity assay demonstrated that all compositions were able to significantly inhibit DPPH mean oxidation. By distinguishing the tested LC-PL-compositions, we determined that compositions I and II were the most effective and composition IV showed the least activity, although its antioxidant capacity was found to be significantly higher than that of the nonformulated PL extract (CE).

### 2.6. Lipid Peroxidation (MDA) Assay

**Figure 7:**
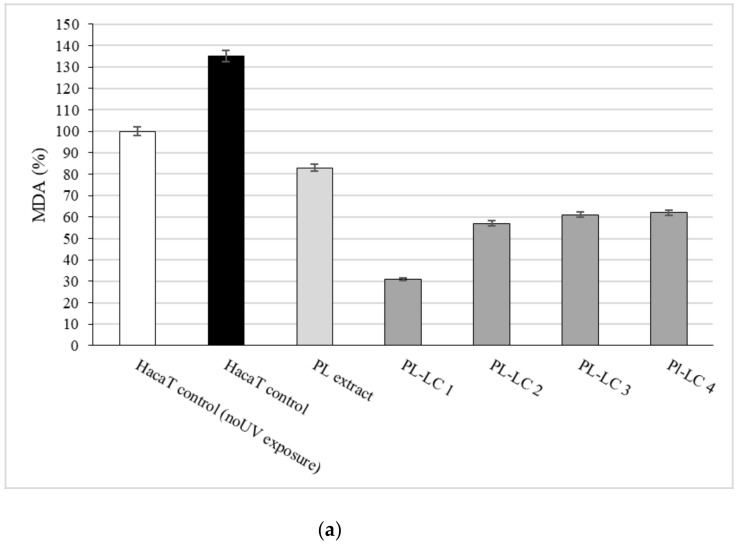

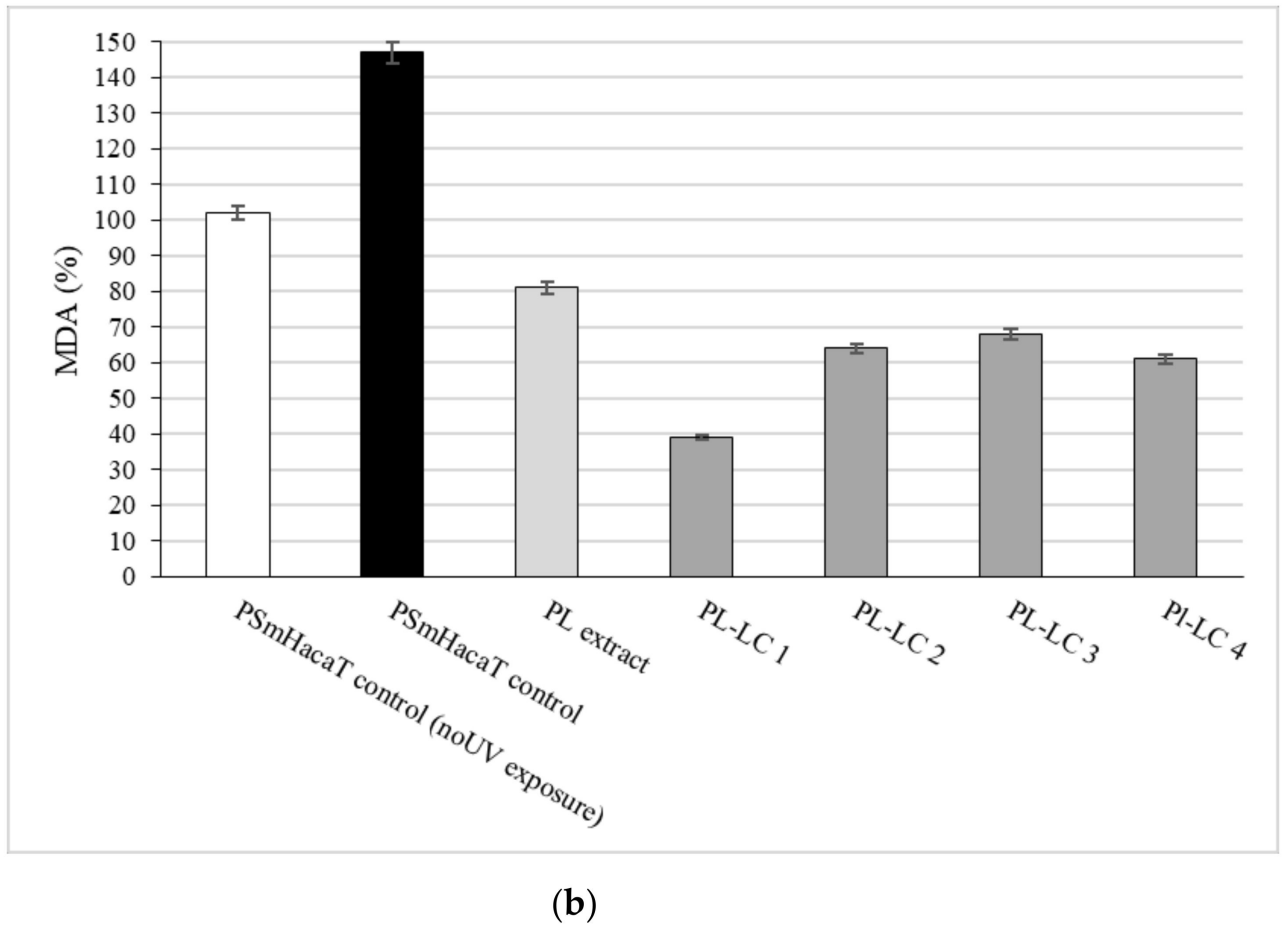
Effect of LC-PL-compositions on HaCaT (**a**) and PSmHaCaT (**b**) MDA levels after 6 min of UV exposure. The positive control PL extract was water. The negative controls were the untreated cells and UV-induced untreated cells. Values are expressed as means ± SD, *n* = 5. Significant differences between LC-PL-composition groups and the control group are marked with an asterisk.

The protective effects of PL-LC compositions I–IV against UV irradiation were examined on HaCaT and PSmHaCaT cell lines. The results of the in vitro antioxidant activity measurements are presented in Figure 7. Evaluated MDA level differences confirmed not only the efficiency of PL extract as an antioxidant complex, but also the importance of the appropriate components of the formulation. Besides all PL-LC compositions being able to decrease MDA levels compared to the untreated and unformulated PL extract controls, we found that PL-LC composition I (with the highest penetration enhancer content) significantly decreased the MDA level.

### 2.7. UV-C Exposure on HaCaT Cells

**Figure 8:**
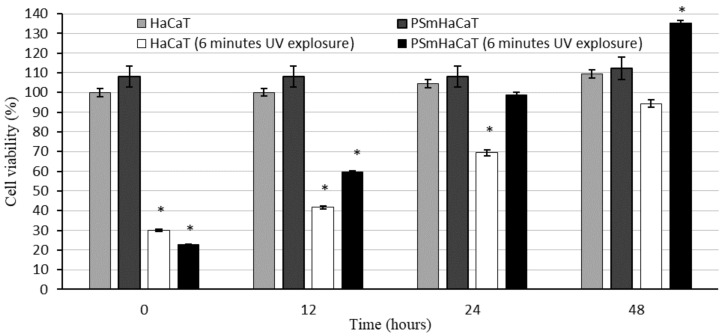
Cell viability evaluation with 3-(4,5-dimethylthiazol-2-yl)-2,5-diphenyltetrazolium bromide (MTT) assay on HaCaT and PSmHaCaT cells after 6 min of UV exposure and their recovery. Each data point represents the mean ± SD, *n* = 5. * means the data is significant since *p* value is 0.01 to 0.05.

**Figure 9:**
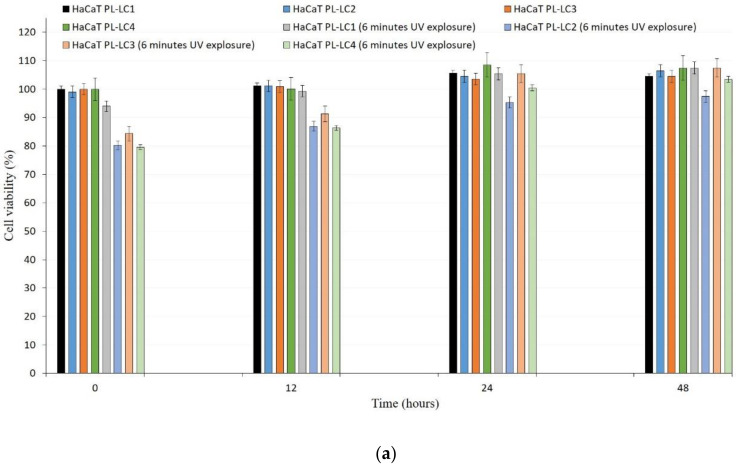

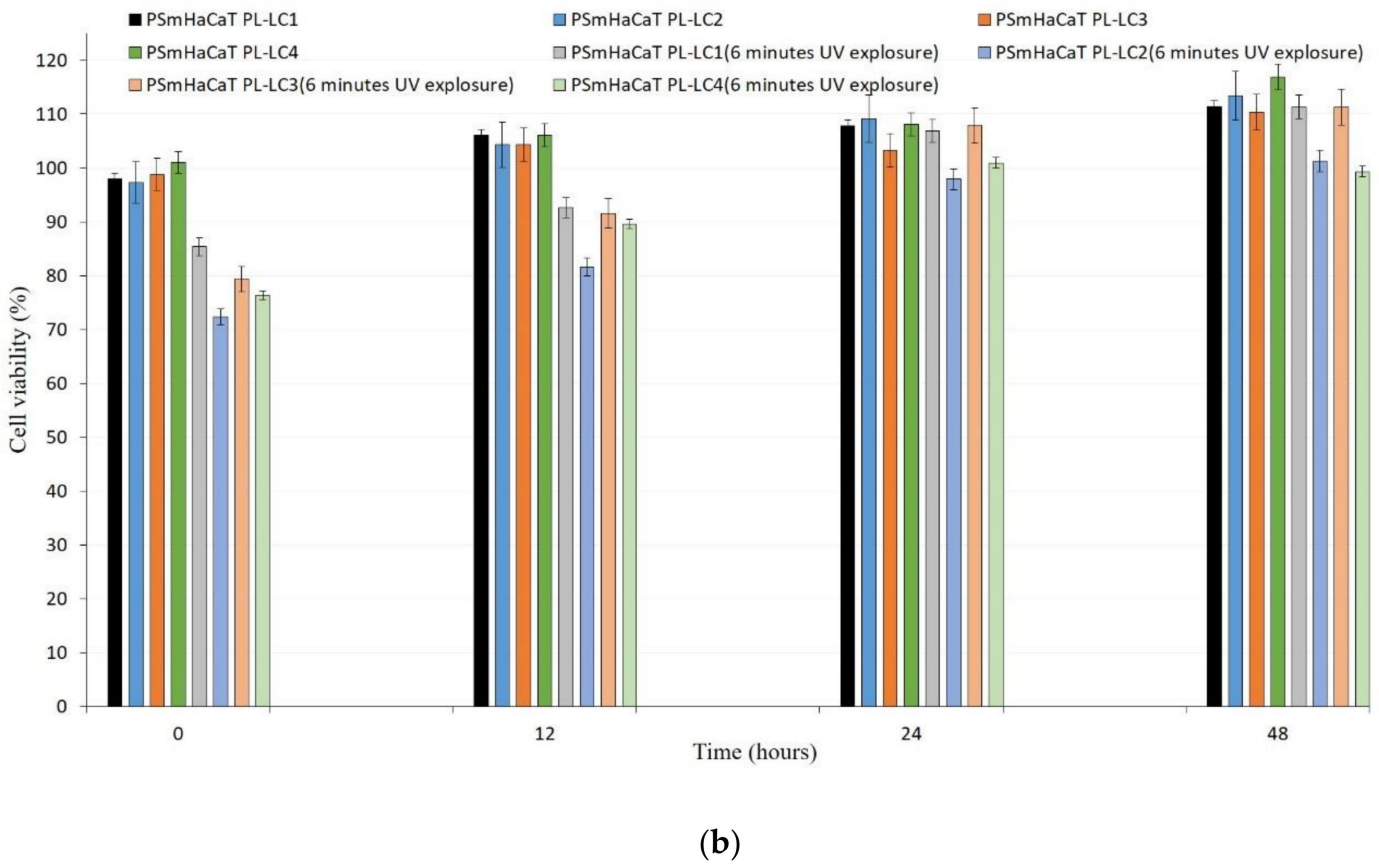
Cell viability evaluation with MTT assay on PL-LC 1–4) pretreated HaCaT (**a**) and PSmHaCaT (**b**) cells after 6 min of UV exposure and their recovery. Each data point represents the mean ± SD, *n* = 5.

The effect of LC-PL compositions against UV-C irradiation was determined on HaCaT and stimulated keratinocyte (PSmHaCaT) cell lines. Compositions were changed by replacing water with an equal volume of Dulbecco’s modified eagle’s medium (DMEM) cell culture medium (Table 2) to avoid the adverse effects of water. Figure 8 shows the cell viability after UV-induced oxidative stress. As in the first experiment, no PL-LC treatment was applied. These results demonstrated the destructive effect of UV-C radiation on the different cell lines and revealed that 6 min of UV-C exposure resulted in a significant decrease in viability. To evaluate the UV-C-stimulated proliferation changes, after the UV-C exposure, MTT follow-up measurements were performed at the 12, 24, and 48 h time points. We concluded that 6 min of UV-C exposure resulted in robust PSmHaCaT cell proliferation. After two days, cell viability of UV-C-stimulated PSmHaCaT cells transcended the viability of untreated cells. Figure 9 shows the cell viability of the PL-LC treated cells after 6 min of UV-induced oxidative stress. Data evaluation showed that PL-LC treatment partly blocked the harmful effect of UV-C radiation. Besides the antioxidant effect of PL extracts, the penetration-enhancing effect of Labrasol/Lauroglycol 90 prevailed as a larger amount of drug entered the cells per unit time from compositions I and III containing more of the surfactant pair, thus retaining cell viability. Comprehensive analysis of follow-up experiments demonstrated that LC-PL treatment was not able to protect the keratinocytes, but the observed hyperproliferation of PSmHaCaT cells caused by UV-C exposure was avoided.

## 3. Discussion

The importance of the role of phytopharmacons in antioxidant therapy continues to increase [28]. Although their advantageous use is proven, the effectiveness of these compounds needs to be improved by pharmaceutical formulation techniques [29].

The usefulness of liquid crystals (LCs) in topical formulations was previously described [30]. LCs are molecules with noncovalent bonds with various phases (mesophase) and different orientations of molecules [15]. LCs are anisotropic materials that demonstrate both liquid- and solid-like properties and the capability to solubilize both oil- and water-soluble compounds [16]. Moreover, LC compositions can be synthesized to form valuable surfactants with a defined penetration enhancer effect [31]. Due to these properties, LC systems are advantageous not only from the formulation point of view, but also for patients in their everyday lives. In the present study, different topical formulations of *Plantago lanceolata* leaf extract were prepared and investigated. We concluded that the choice of suitable excipients and surfactants is essential in the formulation and that the objectives of the study were achieved.

Formulated liquid crystal compositions with an appropriate ratio of Labrasol/Lauroglycol 90 (6:1)/Gelucire 44/14/water were selected using the structured pseudoternary phase diagram, and the percentage of diffused PL extract was evaluated. To evaluate the behavior of the formulated LC systems, a wide variety of in vitro cell line assessments were performed. Besides the HaCaT cell line, TNF-α- and Il-1β-induced human PSmHaCaT cells were used. Impedance-based determination of HaCaT cell proliferation demonstrated an obvious increase in the cell index in each case. Moreover, the kinetic of the proliferation showed differences, i.e., whereas the native HaCaT cells reached the confluent monolayer state after seven days, the IL-1β- and TNF-α-stimulated keratinocytes (PSmHaCaT) reached the plateau phase earlier after rapid proliferation. Furthermore, PSmHaCaT keratinocytes demonstrated higher cell index values at this phase. The performed MTT tests confirmed that neither the formulated LC systems nor their components considerably affected cell viability. Thus, all the formulated compositions were safe. The radical scavenging activity assay demonstrated that the formulated compositions were able to significantly inhibit mean DPPH oxidation. By distinguishing the tested LC-PL compositions, compositions I and II proved to be the most effective and composition IV showed the least activity. The antioxidant properties of the PL-LC systems were evaluated with an MDA lipid peroxidation assay as well. Besides all the PL-LC compositions being able to decrease MDA levels compared to the untreated and unformulated PL extract controls, it was revealed that PL-LC composition I significantly decreased the MDA level. These results agree with the conjecture that penetration enhancers play a role in antioxidant efficiency. This observation was confirmed by the results of the performed UV-C irradiation assessments. Our results demonstrated the destructive effect of UV-C radiation on the different cell lines. The assessment revealed that six minutes of UV-C exposure resulted in a significant viability decrease. To broaden our knowledge after the UV-C exposure, MTT follow-up measurements were performed. These results showed that six minutes of UV-C exposure resulted in robust PSmHaCaT cell proliferation. After two days, the cell viability of UV-C-stimulated PSmHaCaT cells exceeded that of untreated cells. PL-LC treatment unequivocally partly blocked the harmful effect of UV-C radiation. Besides the antioxidant effect of PL extracts, the penetration-enhancing effect of Labrasol/Lauroglycol 90 prevailed as a larger amount of drug entered the cells per unit time from compositions I and III containing more of the surfactant pair, thus retaining cell viability. Moreover, it was revealed that LC-PL treatment was not even able to protect the keratinocytes, but the observed hyperproliferation of PSmHaCaT cells caused by UV-C exposure was avoided. Our findings highlight the importance of antioxidant therapy, especially in the case of inflamed conditions. Our results provide useful data for further formulation procedures to develop innovative, more effective, and safe compositions for topical application.

## 4. Materials and Methods

### 4.1. Materials

*Plantago lanceolata* extract was provided by Gábor Vasas et al. Gelucire 44/14, Labrasol, and Lauroglycol 90 were kind gifts from Gattefossé (Lyon, France). 3-(4,5-Dimethylthiazol-2-yl)-2,5-diphenyltetrazolium bromide (MTT), Dulbecco’s modified Eagle’s medium (DMEM), phosphate-buffered saline (PBS), trypsin- Ethylenediaminetetraacetic acid (EDTA), heat-inactivated fetal bovine serum (FBS), L-glutamine, nonessential amino acids solution, and penicillin–streptomycin were obtained from Sigma-Aldrich (St. Louis, MO, USA). We purchased 96-well cell plates and culturing flasks from VWR International (Debrecen, Hungary). The HaCaT cell line (human keratinocyte cells) was obtained from Cell Lines Service (CLS, Heidelberg, Germany). A Thiobarbituric acid reactive substances (TBARS) Assay Kit was bought from Cayman Chemical (Ann Arbor, MI, USA). 2,2-diphenyl-1-picrylhydrazyl (DPPH), absolute ethanol, IL-1β, and TNF-α were purchased from Sigma-Aldrich (St. Louis, MO, USA).

### 4.2. Dry Plantago lanceolata Leaf Methanolic Extract

Pharmacopoeial standard quality *P. lanceolata* leaves were purchased from commercial origin. Leaves were grinded and reduced to a finely divided powder for further application. The powder was extracted with MeOH under reflux (100 g Dry Weight DW- 400 mL MeOH) for 30 min, filtered, and evaporated to dryness in a rotary evaporator. The extract was defatted with hexane (3 × 50 mL). After the procedure, 100 g of plant drug gave 25.2 g PL extract. The extract was chemically characterized with the LC-ESI-MS method described in [19], after calibration with authentic standards. The extract contained 5.99% acteoside, 2.34% aucubin, and 1.21% catalpol, equivalent to 1.51%, 0.59%, and 0.31% on plant material dry weight, respectively.

### 4.3. Formulation of Liquid Crystals

To obtain the optimal concentration range of components for the boundary of liquid crystals, the pseudoternary phase diagram was constructed using the water dilution method [15]. According to the toxicity screening evaluation, Labrasol, Lauroglycol 90, and Gelucire 44/14 were selected as surfactants. First, the required amount of *Plantago lanceolata* extract (equal to 5% for each composition) was dissolved in the mixture of the surfactants. Then, water was added to the system under moderate stirring. The water content of the compositions varied from 37% to 56%, while the ratio of Labrasol/Lauroglycol 90 was constant (6:1). Four different formulations were selected for further investigation according to the pseudoternary phase diagram (Table 1).

### 4.4. In Vitro Permeation Study

The dissolution rate of *Plantago lanceolata* extract from the formulated LC systems (PL-LC compositions I–IV) was evaluated using a vertical Franz diffusion chamber apparatus (Hanson Microette TM Topical and Transdermal Diffusion Cell System) in triplicate [32]. Samples were applied as the donor phase on a synthetic cellulose acetate membrane previously soaked in isopropyl myristate [33]. We used 30% (*v/v*) alcohol as the receptor phase and samples were stirred with a magnetic rotor at 350 rpm to enhance the dissolution rate of PL extract. The receptor solution was thermostated at 32 °C during the entire experiment to imitate the temperature of physiological skin on the Franz cell membrane. Samples of 1.0 mL of the receptor medium were sequentially collected at predetermined time points (every 15 min for 6 h) and replaced with fresh receptor solution after each sampling. Drug content was analyzed using a UV–Vis spectrophotometer (Shimadzu, Tokyo, Japan) at a wavelength of 517 nm. A calibration curve was determined before the spectroscopic measurements.

### 4.5. Cell Culturing

HaCaT cells were maintained in Dulbecco’s modified Eagle’s medium (DMEM) in a plastic cell culture flask and supplemented with 2 mM L-glutamine, 100 mg/L gentamycin, and 10% heat inactivated fetal bovine serum at 37 °C in 5% CO_2_ atmosphere. The culture medium was changed twice per week. The cells were routinely maintained by regular passaging. The cells used for cytotoxic and antioxidant experiments were between passage numbers 10 and 30. Keratinocytes were treated with a combination of the proinflammatory cytokines TNF-α (20 ng/mL) and IL-1β (25 ng/mL) according to Da Hee Choi et al. [26] to obtain TNF-α-/Il-1β-induced human PSmHaCaT cells.

### 4.6. MTT Viability Assay

The cytotoxicities of applied excipients were evaluated using the MTT (3-(4,5-dimethylthiazol-2-yl)-2,5-diphenyltetrazolium bromide) cell viability assay. HaCaT cells in complete medium were seeded on flat bottom 96-well tissue culture plate at a final density of 10^4^ cells/well. After 7 days, the medium was removed, the cells were washed with PBS, surfactant solutions were added, and the cells were incubated for a further 60 min. After removing the samples, a 3 h incubation with 100 µL MTT solution followed. The dark blue formazan crystals were dissolved in acidic isopropanol (isopropanol:hydrochloride acid = 25:1). The absorbance was measured at 570 nm against a 690 nm reference with FLUOstar OPTIMA Microplate Reader. Cell viability was expressed as the percentage of the untreated control.

### 4.7. Real-Time Cell Analysis and Transepithelial Electrical Resistance Measurements

Transepithelial electrical resistance (TEER) is a well-known and valuable method for in vitro barrier tissue integrity assessment. The measurement concept is relatively straightforward, with TEER measurements performed by applying an AC electrical signal across electrodes placed on both sides of a cellular monolayer and measuring voltage and current to calculate the electrical resistance of the barrier. In our investigation, 10^4^ cells were seeded on special e-microplates as DMEM culturing medium was applied. For the PSmHaCaT cells, inflammation was induced by IL-1β and TNF-α blends, as described previously. Resistance values were measured using the xCELLigence RTCA instrument and data, such as the cell index, were automatically analyzed by the Integrated RTCA software. Observations were confirmed by traditional TEER measurements as well. Transepithelial electrical resistance (TEER) was analyzed by seeding HaCaT and PSmHaCaT cells on 12-well Transwell inserts (Corning Transwell Clear, diameter: 6.5 mm, pore size: 3.0 lm) at a density of 2 × 10^4^ cells/500 µL DMEM [34]. TEER values were measured after 6 h of seeding and then measured every 12 h for 7 days with a Millipore Millicell-Ers 00.001 apparatus.

### 4.8. UV-C Exposure on HaCaT Cells

The UV protective effects of PL-LC compositions were investigated in HaCaT cells [35]. Cells were maintained under the described conditions. HaCaT and PSmHaCaT cells were seeded at the density of 10^4^/200 µL on 96-well plastic plates. The test was performed on the 5th day of their proliferation (when the cells did not reach the total confluency state according to our RTCA measurements). The compositions were changed by replacing water with an equal volume of DMEM cell culture medium to avoid the harmful effect of the water. Each cell line was tested with and without the compositions by UV exposition performed by Oriel Sol-UV-4 UV Solar Simulator (Newport, Irvine, CA, USA). For the first experiment, we worked with the PL-LC formulations. In this experiment, we had two groups of HaCaT and PSmHaCaT cell lines. We applied the UV stimulus only to the second group. As a follow-up, all cell groups were tested directly after the exposure (or after the same six minutes later in case of control group), then 12, 24, and 48 h later via the MTT cell viability assay. In the second part of this experiment, cells were incubated with 0.5% *v/v* PL-LC compositions for 24 h (from their 4th day in proliferation), then exposed to UV-induced oxidative stress for 6 min. HaCaT cell viability was measured directly after the exposure by MTT assay. The follow-up investigation was carried out for 48 h, as in the previous case.

### 4.9. DPPH Radical Scavenging Activity

A DPPH reduction assay was performed to screen the scavenger activity of the liquid crystal formulations containing *Plantago lanceolata*. DPPH is a stable free radical that is capable of changing color in the presence of different antioxidants. The control solutions were the appropriate blank compositions and a 5% *v/v* water dispersion of PL extract (CE). Each sample containing 5% *v/v* PL extract was reacted with the stable DPPH radical in ethanol (96%). The reaction mixture consisted of adding 100 µL of sample, 900 µL of absolute ethanol, and 2 mL of DPPH radical solution (0.06 mM) in absolute ethanol. The mixtures were incubated for 30 min. When DPPH reacted with an antioxidant compound that can donate hydrogen, it was reduced. The reaction resulted in a color change from deep violet to light yellow. Quantitative measurement of the remaining DPPH was carried out with a UV spectrophotometer (Shimadzu Spectrophotometer, Tokyo, Japan) at a wavelength λ of 517 nm. In the case of photometric determination mixtures, absolute ethanol served as the background. The control solutions were the same compositions without *P. lanceolata* extract. To demonstrate the improved antioxidant effect of the combinations, blank *P. lanceolata* extract (5 *v/v%*) was applied as well. The scavenging activity percentage (AA% = antioxidant activity) was determined according to Mensor et al. [36]:

AA% = 100 − [((Abs_sample_ − Abs_blank_) × 100)/Abs_control_].

### 4.10. Lipid Peroxidation (MDA) Assay

Reactive oxygen species can generate the lipid peroxidation process in an organism. Malondialdehyde (MDA) is one of the final products of polyunsaturated fatty acids peroxidation in the cells. An increase in free radicals causes overproduction of MDA. In the lipid peroxidation assay protocol, the MDA in the sample reacts with thiobarbituric acid (TBA) to generate an MDA–TBA adduct. Cells (1 × 10^6^) were homogenized on ice in 300 µL of MDA lysis buffer (with 3 μL BHT (100×), then centrifuged (13,000× *g*, 10 min) to remove insoluble material. An aliquot of 200 μL of the supernatant was analyzed for lipid peroxidation with a TBARS Assay Kit (Cayman Chemical). Lipid peroxidation was determined through the reaction of malondialdehyde (MDA) and thiobarbituric acid (TBA), forming a colorimetric (532 nm) MDA–TBA adduct.

### 4.11. Statistical Analysis

Data were handled and analyzed using Microsoft Excel 2013 and SigmaStat 4.0 (version 3.1; SPSS, Chicago, IL, USA, 2015), and herein presented as means ± SD. The comparison of the results of MTT cell viability assays, free radical scavenging activity test, and in vitro dissolution test was performed with one-way ANOVA and repeated-measures ANOVA followed by Tukey’s or Dunnett post-testing. Differences in means were regarded as significant at *p* < 0.05. All experiments were carried out in quintuplicate and repeated at least five times.

## 5. Conclusions

In our present study, topical PL-LC compositions were designed. According to the results, we found that penetration enhancers played an essential role in improving efficiency. Correlations between the UV irradiation effect causing alterations and inflamed conditions were assessed using the HaCaT cell line. We also found that the formulated PL-LC compositions provided a beneficial option to protect the skin. Our results provide useful data for further investigation.

## Figures and Tables

**Table 1:** Selected compositions of liquid crystals according to the pseudoternary phase diagram.

PL-LC Composition	Plantago Extract	Gelucire 44/14	Labrasol/Lauroglycol 90	Water
1	1.25 mL (4.95%)	10 mL (39.60%)	2.5 mL (9.90%)	11.5 mL (45.55%)
2	1.6 mL (4.92%)	10 mL (31.15%)	2.5 mL (7.79%)	18 mL (56.08%)
3	1.1 mL (5.09%)	11.25 mL (52.08%)	1.25 mL (5.79%)	8 mL (37.04%)
4	1.5 mL (5%)	11.25 mL (37.50%)	1.25 mL (4.17%)	16 mL (53.33%)

**Table 2:** Modified compositions of liquid crystals using Dulbecco’s modified eagle’s medium (DMEM) instead of water.

PL-LC Composition	Plantago Extract	Gelucire 44/14	Labrasol/Lauroglycol 90	DMEM
1	1.25 mL (4.95%)	10 mL (39.60%)	2.5 mL (9.90%)	11.5 mL (45.55%)
2	1.6 mL (4.92%)	10 mL (31.15%)	2.5 mL (7.79%)	18 mL (56.08%)
3	1.1 mL (5.09%)	11.25 mL (52.08%)	1.25 mL (5.79%)	8 mL (37.04%)
4	1.5 mL (5%)	11.25 mL (37.50%)	1.25 mL (4.17%)	16 mL (53.33%)

## Data Availability

Data are available from the corresponding author with the permission of the head of the department. The data that support the findings of this study are available from the corresponding author, [ujhelyi.zoltan@pharm.unideb.hu], upon reasonable request.

## Sample Availability

Samples of the compounds are not available from the authors.
